# The cascade of hypertension prevalence, awareness, treatment, and control in urban-poor communities in Accra, Ghana: a population-based household survey

**DOI:** 10.1186/s12889-025-25409-x

**Published:** 2025-12-23

**Authors:** Olutobi Adekunle Sanuade, Leonard Baatiema, Irene Akwo Kretchy, Lydia Okoibhole, Sandra Boatemaa Kushitor, Carlos S. Grijalva-Eternod, Swaib Abubaker Lule, Daniel Arhinful, Kwadwo Koram, Edward Fottrell

**Affiliations:** 1 Department of Population Health Sciences, Division of Health System Innovation and Research, Spencer Fox Eccles School of Medicine at the University of Utah, Salt Lake City, United States of America; 2 Department of Health Policy, Planning and Management, School of Public Health, University of Ghana, Accra, Ghana; 3 Department of Pharmacy Practice and Clinical Pharmacy, School of Pharmacy, University of Ghana, Accra, Ghana; 4 Institute for Global Health, University College London, London, United Kingdom; 5 Department of Community Health, Ensign Global College, Kpong, Ghana; 6 London School of Hygiene and Tropical Medicine, London, United Kingdom; 7 Noguchi Memorial Institute for Medical Research, University of Ghana, Accra, Ghana

**Keywords:** Hypertension, High blood pressure, Prehypertension, Hypertension control, Hypertension treatment, Survey, Urban-poor, Ga mashie, Ghana

## Abstract

**Background:**

Hypertension is the leading modifiable risk factor for morbidity and mortality from cardiovascular diseases and poses a significant health challenge in low- and middle-income settings, including in sub-Saharan Africa. We set out to: (1) examine the prevalence, awareness, treatment and control of hypertension, and their associated factors, and (2) determine the prevalence of pre-hypertension and its associated factors in an urban poor setting in Ghana.

**Methods:**

This population-based household survey was conducted among a random sample of 854 participants aged ≥ 25 years in Ga Mashie, Accra. Blood pressure was measured using standard protocol. Data were analyzed using multivariable binary logistic regression. Results were presented as adjusted odds ratios (AOR) and their associated 95% confidence intervals (CI).

**Results:**

Of the 854 participants (64.1% women; median age [IQR], 46.0 [14.0] years), 47.3% (95% CI: 44.0%-50.7%) had hypertension, of whom 45.5% (95% CI: 40.7–50.4) were aware, 36.1% (95% CI: 31.6–41.0) received treatment in the last 12 months, 30.0% (95% CI: 25.7–34.6) were on treatment in the last 14 days and 16.1% (95% CI: 12.8–20.0) achieved adequate blood pressure control. Being overweight/obese (AOR = 1.41, 95% CI = 1.01–1.96) and having a family history of diabetes (AOR = 2.03, 95% CI = 1.24–3.33) were associated with hypertension prevalence. Those with diabetes (AOR = 2.95, 95% CI = 1.18–7.38) and current alcohol consumers (AOR = 2.63, 95% CI = 1.15–7.38) were more likely to have controlled hypertension compared to their counterparts. Pre-hypertension prevalence was 27.8%, and the determinants include being male, being employed and having a high household size.

**Conclusions:**

Although high rates of hypertension and prehypertension were reported in this study, awareness, treatment, and control rates were relatively low. These findings suggest that adults in Ga Mashie run a high risk of hypertension complications such as cardiovascular diseases and stroke, and this calls for urgent interventions to reduce blood pressure levels and exposure to hypertension risks in the community.

**Supplementary Information:**

The online version contains supplementary material available at 10.1186/s12889-025-25409-x.

## Background

Despite major advancements in clinical and non-clinical interventions over the past decade, hypertension is still a major public health threat globally. It is the leading risk factor for cardiovascular diseases (CVDs) globally [1]. The Global Burden of Diseases report in 2019 indicates that the absolute number of people aged 30–79 years with hypertension doubled from 331 million women and 317 million men in 1990 to 626 million women and 652 million men in 2019, and this was attributed to population growth and aging [2]. The report revealed that deaths caused by hypertension-related CVDs were 640,239 in young adults, marking a 43% increase from 1990 [3]. However, the number of people living with hypertension is predicted to rise to about 1.5 billion by 2025 [2].

The increasing prevalence of hypertension is principally driven by population aging, urbanization, increasingly unhealthy diets and sedentary lifestyles [2]. Although hypertension is a major public health concern, evidence suggests that low-middle-income countries (LMICs), especially those from Asia and Sub-Saharan Africa, bear the brunt of this burden [4]. In high income countries (HICs), a substantial decline in the prevalence of hypertension has been recorded since the 1970s [1]. Conversely, LMICs have witnessed a rapid rise in the burden of hypertension over the same period [1]. Further, efforts to detect, manage and control hypertension have also seen substantial variations across countries [5]. Even within HICs, control levels for hypertension remain variable and reports from the USA suggest low control levels among African American populations [6]. Evidence from the Global Burden of Diseases showed that although control rates have improved since 1990, these improvements were recorded largely from HICs, whereas LMICs, including those from sub-Saharan Africa (SSA), and parts of South-East Asia still have treatment and control rates of < 25% for women and < 20% for men [2].

Similarly, the burden of hypertension is on the rise in Ghana. Studies to date have reported a prevalence of hypertension ranging from 2.8% to 67.5% [7], and 28.3% from an earlier study [8]. Studies have shown that the rising burden of hypertension in Ghana is largely driven by rapid urbanization, aging, and unhealthy lifestyles [9]. Despite these high burdens, control of high blood pressure (BP) is low, and awareness and treatment of hypertension and the associated risk factors remain poor [10, 11]. There is also limited understanding on hypertension control cascade (i.e., the number of people who drop out across all steps of hypertension prevalence, awareness, treatment, and control) as well as the factors associated with this cascade.

Considering the above context and coupled with the rising burden of hypertension in Ghana and other LMICs, it is important to understand the cascade of hypertension prevalence, awareness, treatment, and control and explore the factors associated with this drop-out. As part of the wider CARE (Contextual Awareness, Response & Evaluation) Diabetes study, this paper describes the cascade of hypertension prevalence, awareness, treatment, and control in Ga Mashie (a cluster of urban-poor communities in Accra, Ghana) and explores the factors associated with the drop-out across all steps of the hypertension cascade of care. The study also examines the prevalence and factors associated with prehypertension in Ga Mashie. Our findings provide insights into contextual factors to target to improve the number of people in each step of the hypertension cascade in Ga Mashie.

## Methods

### Study design and setting

**Fig. 1:**
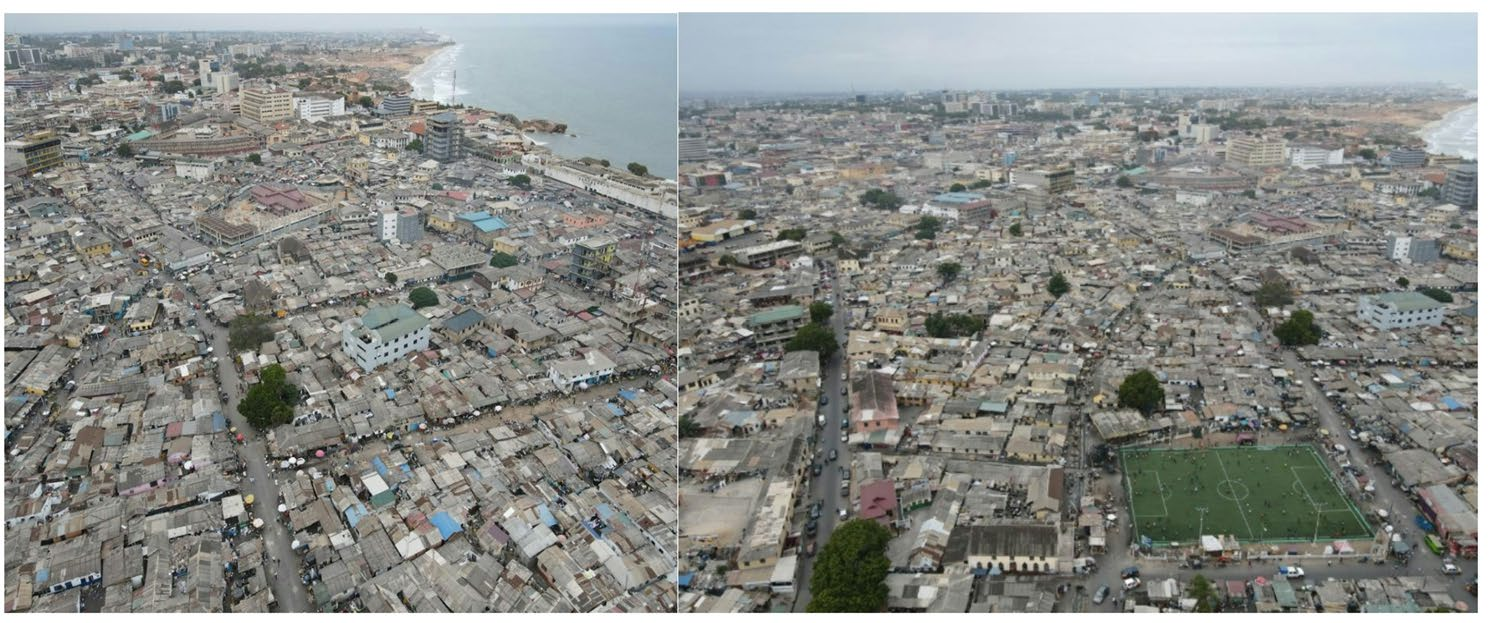
Aerial view of Ga Mashie

This was a cross-sectional population-based study. The study was conducted in Ga Mashie, a cluster of urban-poor communities in Accra. Ga Mashie is a densely populated urban poor community with low socioeconomic status, low literacy rates, poor sanitary conditions, and predominantly old housing structures [12]. Ga Mashie comprises James Town and Ussher Town and it is located east of the Korle Lagoon, on the southwest coast of Accra. It covers an area of 100 hectares along with an estimated population of 125,000 individuals [13]. The area has several formal and informal healthcare facilities and providers, including Ussher Town Polyclinic, a government facility [14]. The community of Ga Mashie consists mainly of the indigenous Ga people and internal migrant populations from other regions in Ghana [13]. Figure 1 shows the aerial view of Ga Mashie. Fishing remains a major source of livelihood, though small-scale trading and some commercial activities now dominate the community [13, 15]. London and Salaga markets serve the area, and these markets are important for trade in a wider variety of local food and non-food items. Street trading is common and often extends to the homes. Food vending is commonly engaged in by most households and Kenkey (a staple dish) is sold in the front yards of many homes [13].

The survey sought to recruit and enroll all individuals aged ≥ 25 years who were residents (someone who has lived in a selected household for the past 12 months) of randomly sampled households (*n* = 1,008). The study excluded women who were either pregnant or had given birth within the last six months. Additionally, anyone deemed unable to provide informed consent or complete the survey, such as individuals with impaired mental capacity or who were hearing impaired and unaided, were also excluded.

### Sampling procedure and sample size

**Fig. 2:**
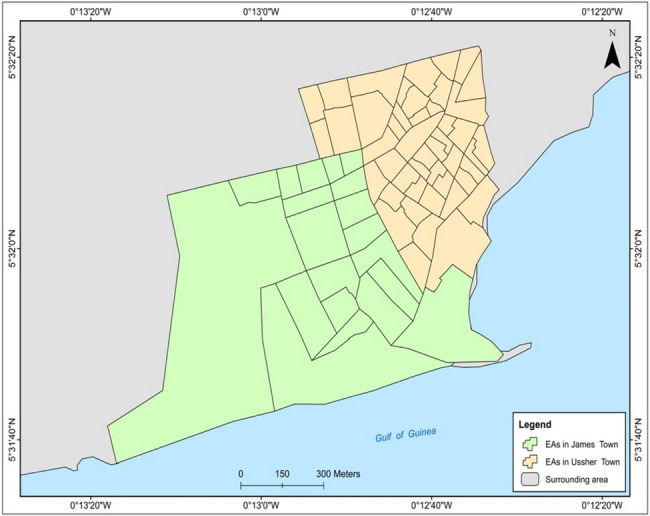
Map of the study area - Enumeration areas

Sample size was calculated in relation to estimating the prevalence of diabetes (a primary goal of the CARE Diabetes project) and we assumed a diabetes prevalence of 5.0%, a precision of 2.0% and a design effect of 2.5. We further assumed an average of two eligible adults per household and a 10% individual refusal to participate. This resulted in a sample of 684 households. However, based on previous field experiences of some of the authors, we further assumed that 40% of households would be empty or non-traceable, leading to an increased sample size of 959 households [16]. We surveyed households from each of the 80 enumeration areas (EAs) of Ga Mashie, to ensure a broad geographical representation. The Ghana Statistical Service (GSS), which conducted the latest census in the area, used simple random sampling within each EA to select a sample of 12 households. As a result, the final sample size increased to 959 households. Figure 2 is a map of the study area showing the EAs. The sampling frame for the survey was all structures and households in Ga Mashie, replicating the approach adopted by GSS in conducting the 2021 Ghana Population and Housing Census [17].

### Training for data collection and pre-testing

The study recruited and trained forty enumerators over a three-day period on survey tools and data collection procedures, including obtaining informed consent, conducting participant interviews, maintaining confidentiality, measuring, and recording anthropometric, blood glucose, and BP data, and using the Open Data Kit (ODK) questionnaires on a mobile device. We developed Standard Operating Procedures (SOPs) with detailed procedures to be followed by the survey researchers and trained enumerators with them. Pre-testing of the survey tools and procedures was conducted among 50 households selected by the study team in the La Municipal area of Accra. Pre-testing assessed all field procedures and data processes, and where necessary modifications were made to address any challenges and maximize data quality in the full survey. Data from the pretest were not included in the final survey data for analysis.

### Data collection

The survey team captured household-level and individual-level data using electronic questionnaires preloaded into ODK Collect on an android mobile device. These mobile devices were encrypted, and password protected. For each household on the sampling list, a unique household identifier was generated, barcoded, and uploaded to ODK. Additional household information including the structure number, house number, participants’ names, telephone number and address were also uploaded to ODK and used to identify a selected household. The household head then confirmed if the information captured about his/her household was correct or not. If correct, the same unique household identifier was applied to all members of the same household. Household and individual-level data were collected separately on different ODK questionnaires for members of the same household. Several quality assurance measures were instituted. For example, to minimize recall bias, collected data were immediately uploaded onto a dedicated secure organizational network analysis (ONA) server, for storage, cleaning, coding, and anonymization. A central coordination unit was set up to upload daily audit data. A standardized survey manual/SOP was developed to guide the data collection process. Households were also systematically mapped to ensure participant variability and enumeration.

### Measurements

Blood pressure measurements: The enumerators measured the BP of each participant following standardized protocols, using an appropriately sized cuff on a digital BP monitor (OMRON - M7 intelli IT HEM-7361T-EBK, Vietnam). We obtained three BP measurements from consenting participants, and used the average of the second and third BP measurements in our analysis [11, 18]. We defined hypertension as self-reported or presenting a systolic BP (SBP) ≥ 140 mmHg, or a diastolic BP (DBP) ≥ 90 mmHg. We defined prehypertension as presenting a SBP of 120–139 mm Hg or a DBP of 80–89 mm Hg [19].

Among those with hypertension: (1) hypertension awareness was present if a participant reported that they had previously been told by a health professional that they had hypertension and duration of hypertension awareness was based on the number of years participants had been diagnosed with hypertension; (2) hypertension treatment in the last 12 months was defined as self-reported use of antihypertensive medications in the last 12 months. Additionally, hypertension treatment was defined as use of antihypertensive medications in the last 14 days prior to the survey, and (3) hypertension control was defined as the proportion of individuals with prior diagnosis of hypertension and with systolic BP < 140 mmHg and diastolic BP of < 90 mmHg in our survey [8, 11, 18].

Socio-demographic variables: These included age, sex (male, female), highest level of education (< secondary, ≥secondary), marital status (single, married), ethnicity (Ga-Dangme, non-Ga-Dangme), employment status (unemployed, employed), and household size. Wealth tertiles were classified into lowest, middle, and highest. The wealth index was constructed from household asset data using principal components analysis. These assets consisted of a television, bicycle, or car, as well as dwelling characteristics such as a source of drinking water, sanitation facilities, and type of flooring material [20].

Lifestyle factors: Physical activity was measured as the number of days respondents spent doing moderate-intensity activities including sports, fitness, or recreational leisure activities, adapted from the Global Physical Activity Questionnaire (GPAQ) [21]. Physical activity was recategorized into two: < 3 days in a week, and ≥ 3 days in a week. We categorized smoking into two: those who had ever smoked (Yes) and those who had never smoked (No). Regarding alcohol intake, we categorized participants into whether they currently consumed alcohol. BMI was calculated as weight (kg) divided by standing height (m2). BMI was then categorized as “not overweight/obese (< 25 kg/m2)” and “overweight/obese (≥ 25kg/m2)”.

Other variables: NCD-Risk is an indicator that reflects dietary risk factors for NCDs, and it is a proxy for consumption of ultra-processed foods [22]. The lower the score, the lower the percentage of dietary energy from ultra-processed foods, and the more recommendations have been met. We used the Diet Quality Questionnaire (DQQ) for Ghana to compute the NCD-Risk scores. The DQQ consisted of 31 items that assessed foods and beverages consumed by participants on the day prior to the survey. The food categories included staples (e.g., bread, rice, waakye, fried rice), vegetables (e.g., carrots, leafy greens, okra), fruits (e.g., mango, orange, papaya), animal-based foods (e.g., eggs, cheese, sausage, beef), and beverages (e.g., condensed milk, soft drinks/soda, coffee). NCD-Risk scores range from 0 to 9; higher scores indicate greater risk for NCDs [22]. Diabetes prevalence was defined as previous diagnosis of diabetes and/or a random glucose concentration of ≥ 11.1 mmol/L [23]. Family history of diabetes was determined as having a member of household with diabetes. Perceived stress was measured using the 10-item Perceived Stress Scale (PSS) with responses varying from 0 (never) to 4 (very often) [24]. Individual scores of PSS range from 0 to 40 with the highest score indicating higher perceived stress [24].

### Data analysis

Descriptive analysis of the study population characteristics was conducted and summarized as frequencies and percentages for categorical variables, means with standard deviation for normally distributed continuous variables, and medians with interquartile ranges (IQR) for non-normally distributed continuous variables. For key proportions related to the hypertension prevalence, awareness, treatment, and control, we reported 95% confidence intervals (CIs) to reflect the precision of our estimates and account for sampling variability. Further, a multivariable logistic regression was conducted using different sub-samples at each stage of the hypertension cascade of care, and we adjusted for the socio-demographic characteristics of the respondents at each stage. In the first stage, we examined the correlates of hypertension prevalence among the study participants (*n* = 854). At the second stage, we assessed factors associated with hypertension awareness among respondents with hypertension (*n* = 404). At the third stage, we examined factors associated with hypertension treatment in the last 12 months among respondents who were aware of their hypertension (*n* = 184). The next stage of the analysis examined factors associated with hypertension treatment in the last 14 days prior to the survey among respondents who were aware of their hypertension and received treatment in the last 12 months before the survey (*n* = 146). The last stage of the hypertension care cascade examined correlates of hypertension control among respondents who were on treatment in the last 14 days prior to the survey (*n* = 121). We also assessed factors associated with pre-hypertension among the study population in our analysis. Results were presented as odds ratios and their associated 95% CIs. Appropriate survey weights were applied before the analysis and adjustment for clustering at EA level was also applied. A two-sided p-value < 0.05 was used to define statistical significance, and the data were analyzed using Stata 14 [25].

## Results

### Characteristics of the study participants

Of the 959 eligible households, 31% (*n* = 301) were not found during the survey period and 1.5% (*n* = 14) refused to participate. Of a total of 1,008 eligible individuals from the 644 households that agreed to take part in the survey, 13.5% (*n* = 136) were not present at home; and 1.8% (*n* = 18) of those present subsequently refused participation in the survey. Thus, our results were based on 854 (84.7%) individuals. Table 1 shows the socio-demographic characteristics of the study population. The median age of the participants was 46.0 years (interquartile range [IQR]: 14.0). Most of the participants were female (64.3%), had less than secondary education (72.5%), single (51.9%), Ga-Dangme (76.9%), employed (73.6%), and overweight/obese (63.4%). Lower proportions of participants had ever smoked (10.6%), were currently consuming alcohol (46.4%), were physically active for ≥ 3 days in a week (7.0%), had diabetes (8.4%), and had family history of diabetes (28.5%). The median household size was 3.0 (IQR:1.6). Participants had a median perceived Stress Score of 18.0 (IQR:0.6), a median NCD Risk Score of 1.0 (IQR: 2.0), mean SBP of 130.7 mm Hg (SD: 28.1), and mean DBP of 84.2 mm Hg (SD: 18.9).

**Table 1:** Characteristics of participants

Variable	*N* = 854	%
Age (years)^a^	854	46.0 (14.0)
Sex Male Female	305 549	35.9 64.1
Highest level of education < Secondary Secondary and above	619 235	72.4 27.6
Marital status^b^ Single Married	442 409	51.7 48.3
Ethnicity Ga-Dangme Non-Ga-Dangme	654 200	76.9 23.1
Employment status Not employed Employed	226 628	26.4 73.6
Ever smoke No Yes	763 91	89.4 10.6
Current alcohol consumption No Yes	458 396	53.6 46.4
Physical activity < 3 days in a week ≥ 3 days in a week	794 60	92.9 7.1
Diabetes No Yes	782 72	91.5 8.5
Overweight/obese No Yes	311 539	36.6 63.4
Wealth tertiles Lowest Middle Highest	277 284 293	32.6 33.2 34.2
Family history of diabetes No Yes	613 241	71.5 28.5
Household size ^a^	854	3.0 (1.6)
Perceived Stress score ^a^	854	18.0 (0.6)
NCD risk score ^a^	854	1.0 (2.0)
SBP (mmHg)^c^	854	130.7 (28.1)
DBP (mmHg)^c^	854	84.2 (18.9)

^a^Median (IQR)

^b^Three individuals have data missing on marital status.

^c^Mean (SD).

SBP- Systolic blood pressure. DBP- Diastolic blood pressure.

### Hypertension prevalence, awareness, treatment, and control

Table 2 provides an overview of hypertension care cascade. The prevalence of hypertension among study participants was 47.3% (95% CI: 44.0%−50.7%). About 46.0% (95% CI: 40.7%−50.4%) of the people with hypertension were aware of their hypertension (i.e., diagnosed by a healthcare professional as having hypertension). Among these patients with hypertension, 36.1% (95% CI: 31.6%−41.0%) had received treatment in the last 12 months, and 30.0% (95% CI: 25.7%−34.6%) had received follow-up treatment in the last 14 days. The results showed that less than one-fifth (16.1%; 95% CI: 12.8%−20.0%) had their BP under control. In addition, the Table shows that the prevalence of pre-hypertension in the study population was 27.8% (95% CI: 24.8%−30.9%).

**Table 2:** Hypertension care cascade

Hypertension status	*N*	% (95% CI)
Pre-hypertension	237	27.8 (24.8–30.9)
Hypertension prevalence	404	47.3 (44.0–50.7.0.7)
Hypertension awareness	184	45.5 (40.7–50.4)
Hypertension treatment in the last 12 months	146	36.1 (31.6–41.0)
Hypertension treatment in the last 14 days	121	30.0 (25.7–34.6)
Hypertension control	65	16.1 (12.8–20.0)

For all variables apart from pre-hypertension and prevalence, the denominator is everyone identified with hypertension. The average duration of awareness was 8.5 (9.7) years

### Factors associated with pre-hypertension

Unadjusted and adjusted odds ratio of factors associated with pre-hypertension and hypertension care cascade are shown in Table 3. The results show that the odds of pre-hypertension reduced with advancement in age (AOR = 0.99, 95% CI = 0.97–0.99). Female respondents were less likely to be pre-hypertensive (AOR = 0.53, 95% CI = 0.36–0.78) compared to male respondents. People who were employed (AOR = 1.99, 95% CI = 1.29–3.07) were more likely to be pre-hypertensive than those who were unemployed. Further, the odds of pre-hypertension increased with household size (AOR = 1.09, 95% CI = 1.01–1.19).

### Factors associated with hypertension prevalence

Odds of hypertension prevalence increased with advancement in age (AOR = 1.05, 95% CI = 1.04–1.07) (Table 3. Although female respondents were more likely to be hypertensive than male respondents (AOR = 1.46, 95% CI = 0.98–2.17), this was not statistically significant. Hypertension prevalence was lower among people who were employed (AOR = 0.61, 95% CI = 0.39–0.93) than those who were unemployed. People who were currently taking alcohol had higher odds of being hypertensive (AOR = 1.66, 95% CI = 1.24–2.23) than people who were not taking alcohol. Furthermore, the odds of hypertension prevalence were higher among people who were overweight/obese (AOR = 1.41, 95% CI = 1.01–1.96) than those who were not.

We performed a sensitivity analysis that defines hypertension only based on our objectively measured BP rather than objective measure or self-report (Supplementary Table 1). Whilst prevalence based on objective measures only was slightly lower (39.7% vs. 47.3%), the direction and magnitude of associations with individual characteristics did not differ substantially.

### Factors associated with hypertension awareness

Among people who were hypertensive, hypertension awareness increased with age (AOR = 1.05, 95% CI = 1.03–1.08) (Table 3). Female respondents were more likely to be aware of their hypertension (AOR = 2.80, 95% CI = 1.41–5.55) than male respondents. Hypertension awareness was higher among respondents who were overweight/obese (AOR = 1.87, 95% CI = 1.11–3.17) than those who were not. Furthermore, the odds of hypertension were higher among those with family history of diabetes (AOR = 2.03, 95% CI = 1.24–3.33) than those without family history of diabetes.

### Factors associated with hypertension treatment in the last 12 months

Among people who were diagnosed with hypertension (i.e., aware of their hypertension), the odds of hypertension treatment in the last 12 months increased with age (AOR = 1.07, 95% CI = 1.01–1.13) (Table 3). Within this group, the odds of receiving treatment in the last 12 months were greater for people in the middle wealth tertiles (AOR = 3.06, 95% CI = 1.16–8.05) and highest wealth tertiles (AOR = 4.51, 95% CI = 1.38–14.71) than people in the lowest wealth tertiles. The odds of receiving treatment in the last 12 months increased with duration of hypertension awareness (AOR = 1.09, 95% CI = 1.04–1.16).

### Factors associated with hypertension treatment in the last 14 days

Among people who were diagnosed with hypertension, the odds of hypertension treatment in the last 14 days increased with age (AOR = 1.10, 95% CI = 1.05–1.15) (Table 3). Within this group, the odds of receiving treatment in the last 14 days were greater for people in the middle wealth tertiles (AOR = 3.06, 95% CI = 1.16–8.05) and highest wealth tertiles (AOR = 4.51, 95% CI = 1.38–14.71) than people in the lowest wealth status. The odds of receiving treatment in the last 14 days increased with duration of hypertension awareness (AOR = 1.09, 95% CI = 1.03–1.14).

### Factors associated with hypertension control

Out of people who were receiving treatment for hypertension in the last 14 days, people who were employed (AOR = 0.34, 95% CI = 0.13–0.89) were less likely to get their hypertension under control than those who were unemployed (Table 3). The odds of achieving hypertension control were higher among people who were currently consuming alcohol (AOR = 2.63, 95% CI = 1.15–7.38) and people with diabetes (AOR = 2.95, 95% CI = 1.18–7.38) than their respective counterparts. 

**Table 3:** Adjusted and unadjusted associations between individual characteristics and outcomes of pre-hypertension and hypertension care cascade

	Prehypertension^a^ (n=854)			Hypertension Prevalence^a^ (n=854)				Hypertension Awareness^b^ (n=404)		
Characteristics	*n*	Unadjusted	Adjusted	*n*	Unadjusted		Adjusted	*n*	Unadjusted	Adjusted
		OR (95% CI)	OR (95% CI)		OR (95% CI)		OR (95% CI)		OR (95% CI)	OR (95% CI)
Age	237	0.98 (0.97–1.00.97.00)**	0.99 (0.97–1.00.97.00)*	404	1.06 (1.05–1.07.05.07)***		1.05 (1.04–1.07.04.07)***	184	1.05 (1.03–1.07.03.07)***	1.05 (1.03–1.08.03.08)***
Level of education										
< Secondary (ref)	166	1	1	311	1		1	142	1	1
Secondary and above	71	1.18 (0.84–1.67.84.67)	1.05 (0.71–1.55.71.55)	93	0.65 (0.50–0.84.50.84)		0.92 (0.64–1.34.64.34)	42	0.98 (0.62–1.55.62.55)	1.25 (0.71–2.22.71.22)
Sex										
Male (ref)	107	1	1	121	1		1	35	1	1
Female	130	0.57 (0.42–0.78.42.78)***	0.53 (0.36–0.78.36.78)**	283	1.61 (1.19–2.19.19.19)**		1.46 (0.98–2.17.98.17)	149	2.73 (1.78–4.20.78.20)***	2.80 (1.41–5.55.41.55)**
Wealth tertiles										
Lowest (ref)	80	1	1	134	1		1	60	1	1
Middle	79	0.95 (0.67–1.35.67.35)	0.85 (0.57–1.27.57.27)	144	1.10 (0.79–1.52.79.52)		1.17 (0.81–1.71.81.71)	76	1.38 (0.89–2.14.89.14)	1.32 (0.76–2.28.76.28)
Highest	78	0.89 (0.57–1.41.57.41)	0.62 (0.37–1.07.37.07)	126	0.81 (0.56–1.16.56.16)		1.27 (0.79–2.04.79.04)	48	0.76 (0.45–1.28.45.28)	0.95 (0.52–1.76.52.76)
Marital status										
Single (ref)	115	1	1	221	1		1	111	1	1
Married	122	1.21 (0.91–1.61.91.61)	0.91 (0.67–1.25.67.25)	180	0.79 (0.61–1.01.61.01)		1.14 (0.85–1.55.85.55)	71	0.65 (0.42–0.98.42.98)*	1.37 (0.77–2.42.77.42)
Ethnicity										
Ga-Dangme (ref)	57	1	1	89	1		1	37	1	1
Non-Ga-Dangme	180	0.95 (0.69–1.32.69.32)	1.01 (0.72–1.43.72.43)	315	1.16 (0.85–1.59.85.59)		0.86 (0.59–1.24.59.24)	147	1.23 (0.74–2.05.74.05)	0.99 (0.56–1.73.56.73)
Employment status										
Not employed (ref)	39	1	1	147	1		1	86	1	1
Employed	198	2.21 (1.51–3.23.51.23)***	1.99 (1.29–3.07.29.07)*	257	0.37 (0.27–0.51.27.51)***		0.61 (0.39–0.93.39.93)*	98	0.44 (0.28–0.69.28.69)***	0.72 (0.38–1.35.38.35)
Ever smoke										
No (ref)	206	1	1	366	1		1	167	1	1
Yes	31	1.40 (0.84–2.31.84.31)	1.17 (0.66–2.07.66.07)	38	0.78 (0.53–1.13.53.13)		0.95 (0.58–1.56.58.56)	17	0.96 (0.46–2.02.46.02)	2.65 (0.98–7.21.98.21)
Current alcohol consumption										
No (ref)	129	1	1	210	1		1	106	1	1
Yes	108	0.96 (0.73–1.25.73.25)	0.75 (0.55–1.02.55.02)	194	1.13 (0.85–1.51.85.51)		1.66 (1.24–2.23.24.23)***	78	0.66 (0.43–1.01.43.01)	0.91 (0.54–1.56.54.56)
Physical activity										
<3 days in a week (ref)	216	1	1	386	1		1	177	1	1
≥3 days in a week	21	1.44 (0.83–2.51.83.51)	1.12 (0.64–1.98.64.98)	18	0.45 (0.25–0.82.25.82)		0.66 (0.33–1.33.33.33)	7	0.75 (0.28–1.97.28.97)	1.29 (0.48–3.48.48.48)
Diabetes										
No (ref)	224	1	1	352	1		1	149	1	1
Yes	13	0.68 (0.32–1.45.32.45)	0.68 (0.32–1.45.32.45)	52	3.18 (1.69–5.99.69.99)		1.68 (0.82–3.42.82.42)	35	2.80 (1.40–5.61.40.61)**	1.96 (0.87–4.40.87.40)
Overweight/obese										
No (ref)	87	1	1	125	1		1	42	1	1
Yes	150	0.99 (0.73–1.34.73.34)	1.30 (0.94–1.80.94.80)	278	1.58 (1.21–2.08.21.08)**		1.41 (1.01–1.96.01.96)*	141	2.03 (1.35–3.06.35.06)*	1.87 (1.11–3.17.11.17)*
Perceived Stress score	237	1.01 (0.98–1.04.98.04)	1.00 (0.97–1.03.97.03)	404	0.99 (0.96–1.02.96.02)		0.99 (0.96–1.03.96.03)	184	1.00 (0.95–1.04.95.04)	1.01 (0.96–1.06.96.06)
NCD risk score		1.01 (0.89–1.15.89.15)	0.97 (0.84–1.12.84.12)	404	0.87 (0.79–0.96.79.96)**		1.00 (0.88–1.14.88.14)	184	1.06 (0.89–1.27.89.27)	1.12 (0.92–1.36.92.36)
Family history of diabetes										
No (ref)	170	1	1	279	1		1	110	1	1
Yes	67	1.00 (0.67–1.50.67.50)	1.18 (0.78–1.77.78.77)	125	1.29 (0.95–1.75.95.75)		1.02 (0.75–1.40.75.40)	74	2.22 (1.38–3.59.38.59)**	2.03 (1.24–3.33.24.33)*
Household size	237	1.09 (1.01–1.19.01.19)*	1.09* (1.01–1.19.01.19)	404	0.94 (0.85–1.04.85.04)		0.96 (0.86–1.06.86.06)		-	-
Duration of hypertension awareness		-	-		-		-		-	-

	Hypertension treatment in the last 12 months^c^ (n=184)			Hypertension treatment in the last 14 days^c^ (n=146)				Hypertension control^d^ (n=121)		
Characteristics	*n*	Unadjusted	Adjusted	*n*		Unadjusted	Adjusted	*n*	Unadjusted	Adjusted
		OR (95% CI)	OR (95% CI)			OR (95% CI)	OR (95% CI)		OR (95% CI)	OR (95% CI)
Age	146	1.07 (1.03–1.13.03.13)***	1.07 (1.01–1.13.01.13)*	121		1.09 (1.05–1.13.05.13)***	1.11 (1.05–1.15.05.15)***	45	1.02 (0.99–1.05.99.05)	1.04 (0.98–1.10.98.10)
Level of education										
< Secondary (ref)	112	1	1	94		1	1	35	1	1
Secondary and above	34	1.14 (0.45–2.89.45.89)	0.87 (0.30–2.55.30.55)	27		0.92 (0.44–1.92.44.92)	0.97 (0.35–2.66.35.66)	10	0.99 (0.49–2.00.49.00)	0.54 (0.19–1.57.19.57)
Sex										
Male (ref)	27	1	1	20		1	1	11	1	1
Female	119	1.18 (0.43–3.22.43.22)	0.99 (0.23–4.33.23.33)	101		1.58 (0.68–3.65.68.65)	1.12 (0.32–3.95.32.95)	34	0.42 (0.19–0.90.19.90)	0.25 (0.06–1.08.06.08)
Wealth tertiles										
Lowest (ref)	43	1	1	34		1	1	11	1	1
Middle	62	1.75 (0.74–4.16.74.16)	3.02 (1.32–6.91.32.91)**	54		1.88 (1.02–3.45.02.45)*	3.06 (1.16–8.05.16.05)*	23	1.55 (0.63–3.81.63.81)	1.29 (0.43–3.90.43.90)
Highest	41	2.31 (0.74–4.16.74.16)	7.74 (1.43–41.97.43.97)*	33		1.68 (0.81–3.49.81.49)	4.51 (1.38–14.71.38.71)*	11	1.04 (0.40–2.76.40.76)	1.05 (0.28–3.92.28.92)
Marital status										
Single (ref)	88	1	1	76		1	1	29	1	1
Married	57	1.06 (0.47–2.43.47.43)	2.07 (0.79–5.41.79.41)	44		0.75 (0.38–1.47.38.47)	1.44 (1.38–14.71.38.71)	15	0.84 (0.36–1.96.36.96)	0.85 (0.21–3.49.21.49)
Ethnicity										
Ga-Dangme (ref)	29	1	1	25		1	1	11	1	1
Non-Ga-Dangme	117	1.08 (0.48–2.40.48.40)	1.30 (0.47–3.78.47.78)	96		0.90 (0.44–1.85.44.85)	0.97 (0.38–2.48.38.48)	34	0.70 (0.27–1.82.27.82)	0.83 (0.27–2.59.27.59)
Employment status										
Not employed (ref)	75	1	1	64		1	1	29	1	1
Employed	71	0.39 (0.19–0.80.19.80)*	0.65 (0.22–1.98.22.98)	57		0.48 (0.25–0.88.25.88)*	1.28 (0.51–3.23.51.23)	16	0.47 (0.21–1.04.21.04)	0.34 (0.13–0.87.13.87)*
Ever smoke										
No (ref)	136	1	1	114		1	1	42	1	1
Yes	10	0.33 (0.12–0.91.12.91)*	0.27 (0.05–1.42.05.42)	7		0.32 (0.11–0.95.11.95)*	0.53 (0.14–2.10.14.10)	3	1.29 (0.34–4.91.34.91)	0.67 (0.08–5.99.08.99)
Current alcohol consumption										
No (ref)	90	1	1	81		1	1	26	1	1
Yes	56	0.45 (0.20–1.03.20.03)	1.77 (0.31–10.28.31.28)	40		0.32 (0.15–0.69.15.69)**	0.56 (0.23–1.33.23.33)	19	1.91 (0.90–4.06.90.06)	2.63 (1.15–6.03.15.03)
Physical activity										
<3 days in a week (ref)	141	1	1	118		1	1	43	1	1
≥3 days in a week	5	0.64 (0.12–3.26.12.26)	1.77 (0.31–10.28.31.28)	3		0.38 (0.81–1.73.81.73)	0.97 (0.20–1.33.20.33)	2	3.49 (0.28–43.08.28.08)	5.26 (0.30–92.47.30.47)
Diabetes										
No (ref)	117	1	1	94		1	1	31	1	1
Yes	29	1.32 (0.49–3.54.49.54)	1.11 (0.36–3.46.36.46)	27		1.97 (0.85–4.55.85.55)	1.38 (0.57–3.33.57.33)	14	2.19 (0.90–5.35.90.35)	2.95 (1.18–7.38.18.38)*
Overweight/obese										
No (ref)	31	1	1	25		1	1	10	1	1
Yes	114	1.50 (0.70–3.19.70.19)	1.34 (0.49–3.69.49.69)	95		1.40 (0.68–2.88.68.88)	1.65 (0.56–4.86.56.86)	35	0.87 (0.36–2.14.36.14)	1.20 (0.36–3.98.36.98)
Perceived Stress score	146	1.00 (0.92–1.08.92.08)	1.04 (0.95–1.14.95.14)	121		1.00 (0.92–1.07.92.07)	1.04 (0.96–1.13.96.13)	45	0.98 (0.89–1.08.89.08)	1.02 (0.92–1.14.92.14)
NCD risk score	146	0.77 (0.55–1.08.55.08)	0.72 (0.48–1.06.48.06)	121		0.78 (0.58–1.06.58.06)	0.78 (0.54–1.13.54.13)	45	0.98 (0.68–1.40.68.40)	0.87 (0.49–1.53.49.53)
Family history of diabetes										
No (ref)	90	1	1	76		1	1	26	1	1
Yes	56	0.69 (0.34–1.40.34.40)	0.62 (0.24–1.56.24.56)	45		0.69 (0.38–1.26.38.26)	0.69 (0.32–1.46.32.46)	19	1.41 (0.63–3.11.63.11)	1.49 (0.59–3.77.59.77)
Household size	146	0.82 (0.67–1.01.67.01)	0.85 (0.66–1.11.66.11)	121		0.89 (0.72–1.09.72.09)	0.97 (0.72–1.29.72.29)	45	1.04 (0.80–1.34.80.34)	1.19 (0.86–1.65.86.65)
Duration of hypertension awareness	146	1.09 (1.03–1.13.03.13)***	1.09 (1.04–1.15.04.15)*	121		1.08 (1.03–1.13.03.13)*	1.09 (1.03–1.14.03.14)**	45	0.97 (0.93–1.01.93.01)	0.96 (0.91–1.01.91.01)

Exponentiated coefficients; 95% confidence intervals in brackets. AOR-Adjusted Odds Ratio

a Adjusted for age, level of education, sex, wealth tertiles, marital status, ethnicity, smoking, alcohol consumption, physical activity, diabetes prevalence, perceived stress score, NCD risk score, family history of diabetes, and household size

b Adjusted for age, level of education, sex, wealth tertiles, marital status, ethnicity, smoking, alcohol consumption, physical activity, diabetes prevalence, perceived stress score, NCD risk score, family history of diabetes

c Adjusted for age, level of education, sex, wealth tertiles, marital status, ethnicity, smoking, alcohol consumption, physical activity, diabetes prevalence, perceived stress score, NCD risk score, family history of diabetes, household size, and duration of hypertension awareness

d Adjusted for age, level of education, sex, wealth tertiles, marital status, ethnicity, smoking, alcohol consumption, physical activity, diabetes prevalence, perceived stress score, NCD risk score, family history of diabetes, household size, and duration of hypertension awareness

* *p* < 0.05, ** *p* < 0.01, *** *p* < 0.001

## Discussion

### Summary of findings

This study estimated prevalence, awareness, treatment, and control of hypertension in Ga Mashie, an urban poor community in Accra, Ghana. Our findings showed that people dropped out across all steps of the hypertension care cascade and significant drops were seen at the awareness, treatment, and control stages. Overall, close to 50% of adults aged ≥ 25 years in Ga Mashie had hypertension. Among respondents with hypertension, about 46% were aware of it, 36% were receiving treatment in the last 12 months, 30% received follow-up treatment in the last 14 days, and only 16% had their BP under control.

### Hypertension prevalence

Compared to a similar study carried out in Ga Mashie over 10 years ago (with a hypertension prevalence of 28.3%) [8], our study showed that hypertension prevalence had significantly increased in this community. The study by Awuah et al. (2014) and our study were both based on a representative sample of Ga Mashie. Nevertheless, while the current study only collected data from individuals aged ≥ 25 years in Ga Mashie, the data by Awuah et al. (2014) was based on individuals aged ≥ 18 years. Additionally, the overall prevalence of hypertension in this study (47.3%) was higher than in previous studies in Accra (28.3%) in 2011 [26] and Kumasi (28.7%) in 2001–2002 [27]. Our study, as well as the study conducted by Awuah et al. (2014) [26], were both conducted in poor urban communities. However, the study conducted by Cappuccio et al. (2004) [27]was conducted in semi-urban and rural villages. This suggests true differences in the prevalence of hypertension between this study and the one conducted by Awuah et al. (2014). The disparities in hypertension prevalence between the study by Cappuccio et al. (2004) and this study could be attributed to differences in the characteristics of the populations studied.

Our study revealed that while hypertension prevalence was higher among older participants, women and people currently taking alcohol, it was lower among people who were employed. Previous studies have shown similar pattern that hypertension prevalence increases with advancement in age [11, 18]. Contrary to our finding, which showed higher odds of hypertension prevalence among women, a study conducted by Awuah et al. (2014) found higher rate of hypertension prevalence among men [8]. This is not surprising because a previous study had predicted an increase in hypertension prevalence among women (13%) than men (9%) between 2000 and 2025 mainly due to aging and longer life expectancies in women than in men [28].

Furthermore, our findings align with a review of population-based studies in Ghana which showed that alcohol consumption is associated with the prevalence of hypertension [29]. Studies conducted outside of Ghana have also demonstrated that a linear relationship exists between alcohol intake and BP both in the short- and long-term [30, 31]. Additionally, we found that hypertension prevalence was lower among those employed compared to those who were unemployed. A meta-analysis has shown that having a lower occupational grade is linked to a higher prevalence or incidence of hypertension [32]. Even though employment has also been linked to lower hypertension prevalence and incidence in high-income countries (HICs), there is less evidence to support this association than there is for wealth and education [33].

### Hypertension awareness

Among people with hypertension, our findings showed that close to half (45.5%) were aware of their condition. We found that hypertension awareness was higher among older adults, women, people who had ever smoked, people who were obese and people with a family history of diabetes. These findings align with the patterns shown by previous studies with respect to the correlates of hypertension awareness [11, 18, 34, 35]. The higher hypertension awareness among women may be attributed to their propensity to seek out healthcare compared to men’s unwillingness to report and seek medical attention [18, 36, 37]. For instance, more Ghanaian women than men have registered with the National Health Insurance Scheme (NHIS) [38, 39], indicating better access to healthcare. Another major finding is that hypertension awareness was higher among people with a family history of diabetes. This could be a positive “enabler” which could be the focus of intervention strategies. Further, the awareness rate in this study is similar to the national estimate based on findings from the 2014 Ghana Demographic and Health Survey (46%) [18]. Even though hypertension awareness has increased in our study community compared to the last 10 years (< 8%) [8], the current level still shows the need to intensify BP screening to identify people unaware of their hypertension in the community.

### Hypertension treatment

The hypertension care cascade analysis revealed that, despite a confirmed diagnosis of hypertension, a significant proportion of participants (64% in the last 12 months and 70% in the last 14 days) were not receiving treatment. Previous research has shown that in general, non-initiation of therapy [40], and lack of motivation for therapy [41] are the two main reasons why hypertension is not currently being treated. These findings from this community have some implications. First, the finding suggests the need to improve access to health insurance in this community to invariably increase access to the antihypertensive medications covered by health insurance. Nevertheless, there is evidence showing that even people with access to health insurance often cannot access antihypertensive medications because of frequent drug stock-outs, indicating that people have to pay for these drugs out-of-pocket [18]. Therefore, it is important to tackle other health systems-related challenges to ensure that people with hypertension (especially those with low socioeconomic status) have access to antihypertensive medications as and when needed [42].

Furthermore, previous studies have reported the possibility of medication non-adherence even when people with hypertension have access to antihypertensive medications [43]; this gap has been associated with uncontrolled BP, poor clinical outcomes [44, 45], and an increased cost to the healthcare system [46]. The level of hypertension treatment in this study also suggests the need to implement strategies to promote medication adherence among people with hypertension in this community. A review of randomized clinical trials showed that interventions such as fixed-dose combination pills to reduce daily pill burden, clinical pharmacist consultation for disease co-management, and medication-taking reminders can enhance adherence [47]. Further studies need to explore and evaluate implementation strategies to promote medication adherence and access to antihypertensive medications in Ga Mashie.

Our findings showed that the odds of hypertension treatment were higher among older adults, people in higher wealth tertiles, people with lower household size and people with higher duration of hypertension awareness. As indicated above, people with hypertension often cannot access antihypertensive medications in the NHIS thus forcing people to pay out-of-pocket. This may be a plausible explanation for higher treatments among people in the higher wealth tertiles because of more access to resources to purchase antihypertensive medications. In addition, higher household size indicates higher household consumption expenditure [48], and this may explain why hypertension treatment was lower among participants in larger households.

### Hypertension control

Our results showed that even though about one-third of our study participants were receiving hypertension treatment in the last 14 days before the survey, less than one-fifth (16.1%) had their BP under control. Poor hypertension control is attributed to a number of health system- and health provider-related problems, including a lack of anti-hypertensive medications, a long travel time to hospitals, excessive drug costs, insufficient counseling, and an absence of the necessary knowledge, skills, and resources to address hypertension [49]. Our findings showed that despite greater hypertension prevalence and awareness among women compared to men, women seemed to drop out of the cascade disproportionately.This may be because men are more likely to have manifest cardiovascular disease, leading to more aggressive antihypertensive treatment among them compared to women [50]. Further, we found that hypertension control was lower among people who were employed but higher among those with diabetes. A plausible explanation for lower hypertension control among employed individuals may be their exposure to high levels of work-related stress [51, 52]. Further, our findings showed that the current intake of alcohol was associated with hypertension control. While this finding is unexpected, previous observational studies have shown that low to moderate alcohol intake may have a cardiovascular protective effect [53]. Additionally, we found that hypertension control was higher among those with diabetes mellitus, and this is contrary to what previous literature has shown. Previous research shows that hypertension control in patients with diabetes presents a great challenge due to lower target BP and poor response to treatment [54, 55]. A higher rate of hypertension control among people with diabetes in our study may be explained by higher hospital visits, which perhaps increase interactions with health professionals and consequently increase medication adherence [45, 56]. Further research is needed to understand the complex interaction between diabetes and hypertension.

### Pre-hypertension

We assessed the prevalence of prehypertension in Ga Mashie to better understand the danger of high BP in this community. Our results showed a relatively high (28%) prevalence of prehypertension among our study participants, and age, sex and employment status were factors associated with prehypertension. According to a recent study, prehypertension is linked to an increased risk of cardiovascular disease (CVD) [57] and chronic kidney diseases [58, 59]. People with prehypertension require targeted individual-level interventions that take into account their age, sex and employment status as well as community-based initiatives that address the social determinants of health- this will not only alleviate the number of people that will develop hypertension in subsequent years but will invariably reduce the burden of cardiovascular and chronic kidney diseases in this community.

#### Limitations

Our study has some limitations. Despite being a representative study of Ga Mashie, the study was cross-sectional, and this limits its ability to provide information on an illness (i.e., hypertension) that is essentially chronic and to draw any conclusions about causal associations. Also, because BP readings were only taken at one-time points and without temperature adjustment, the prevalence of hypertension in the sample may have been underestimated. Cross-sectional data collection has the additional drawbacks that it cannot assess causal associations or trends over time [60]. Furthermore, we did not adjust for multiple outcome testing; however, the interpretation of our findings is based on magnitude, directions, and patterns of associations rather than absolute statistical significance. We also acknowledge the diminishing sample size along the cascade, which may limit statistical power and generalizability, and the potential for over-adjustment, which could affect the robustness of observed associations. However, we do not consider these to be significant weaknesses given the study’s focus and objectives. Finally, the discrepancy in the relationship of the NCD-Risk score with prehypertension, hypertension prevalence, awareness, treatment, and control may be attributed to limitations in dietary assessment tools, which might not fully capture habitual dietary patterns or nutrient intake critical for assessing chronic disease risk [22]. Additionally, cultural dietary practices or food preparation methods, such as the use of high-sodium condiments or cooking technique may influence the risk of hypertension [61] but are not adequately reflected in the NCD-Risk score. Despite these limitations, our study is a large population-based survey with high-quality data which helps to fill an important information gap about the current rates of hypertension care cascade and factors driving this in poor urban communities like Ga Mashie.

## Conclusion

This study provides a comprehensive overview of the gaps in all steps of the continuum of hypertension prevalence, awareness, treatment, and control in Ga Mashie and an in-depth understanding of the characteristics of the people at risk of dropping out at each step of this continuum. While high rates of hypertension and prehypertension were reported in this study, awareness, treatment, and control rates were relatively low. This suggests that adults in Ga Mashie run a high risk of hypertension complications such as CVD and stroke. The alarming rates of prehypertension and hypertension in this community call for urgent interventions to reduce BP levels in the community. Particularly, future research needs to explore and evaluate implementation strategies for the adoption and sustainability of evidence-based interventions for hypertension management and control in this community. This may start by implementing community-based interventions which have been shown to improve hypertension diagnosis, treatment, and control in similar settings as Ga Mashie.

## Supplementary Information

Supplementary Material 1.

## Data Availability

Data analyzed during this study will be made available upon reasonable request to the corresponding author Olutobi A. Sanuade by emailing him at olutobi.sanuade@hsc.utah.edu.
