# Supplementary material for: The cascade of hypertension prevalence, awareness, treatment, and control in urban-poor communities in Accra, Ghana: a population-based household survey
Source: BMC Public Health. 2025 Dec 23;25:4288. doi: 10.1186/s12889-025-25409-x (PMC12729810; doi:10.1186/s12889-025-25409-x)
Supplement: Supplementary file 1 — Supplementary Material 1. [file 12889_2025_25409_MOESM1_ESM.docx]

**Supplementary Table 1.** Associations between individual characteristics and hypertension prevalence based on objective blood pressure measure

| **Characteristics** | **Hypertension (n=339)** | |
| --- | --- | --- |
|  | n | **AOR (95% CI)** |
| **Age** | 339 | 1.04 (1.02-1.05)*** |
| **Level of education** | |  |
| < Secondary (ref) | 264 | 1 |
| Secondary and above | 75 | 0.83 (0.57-1.19) |
| **Sex** |  |  |
| Male (ref) | 104 | 1 |
| Female | 235 | 1.31 (0.89-1.94) |
| **Wealth tertiles** | |  |
| Lowest | 118 | 1 |
| Middle | 114 | 0.98 (0.66-1.45) |
| Highest | 107 | 1.08 (0.69-1.71) |
| **Marital status** | |  |
| Single | 183 | 1 |
| Married | 155 | 1.13 (0.81-1.59) |
| **Ethnicity** |  |  |
| Ga-Dangme | 74 | 1 |
| Non-Ga-Dangme | 265 | 0.92 (0.62-1.36) |
| **Employment status** | |  |
| Not employed | 115 | 1 |
| Employed | 224 | 0.82 (0.52-1.28) |
| **Ever smoke** |  |  |
| No | 308 | 1 |
| Yes | 31 | 0.88 (0.53-1.46) |
| **Current alcohol consumption** | | |
| No | 178 | 1 |
| Yes | 161 | 1.38 (1.03-1.85)* |
| **Physical activity** | |  |
| <3 days in a week | 324 | 1 |
| ≥3 days in a week | 15 | 0.68 (0.34-1.36) |
| **Diabetes** |  |  |
| No | 303 | 1 |
| Yes | 36 | 1.00 (0.55-1.83) |
| **Overweight/obese** | |  |
| No | 108 | 1 |
| Yes | 230 | 1.29 (0.92-1.80) |
| **Perceived Stress score** | 339 | 0.99 (0.96-1.02) |
| **NCD risk score** | 339 | 0.97 (0.85-1.11) |
| **Family history of diabetes** | | |
| No | 244 | 1 |
| Yes | 95 | 0.85 (0.64-1.15) |
| **Household size** | 339 | 0.96 (0.86-1.07) |

Exponentiated coefficients; 95% confidence intervals in brackets. OR=Odds Ratio.

^*^ *p* < 0.05, ^**^ *p* < 0.01, ^***^ *p* < 0.001
